# Inverse Association between High-Density Lipoprotein Cholesterol and Adverse Outcomes among Acute Ischemic Stroke Patients with Diabetes Mellitus

**DOI:** 10.3390/biomedicines9121947

**Published:** 2021-12-20

**Authors:** Guoliang Hu, Yuesong Pan, Mengxing Wang, Xia Meng, Yong Jiang, Zixiao Li, Hao Li, Yongjun Wang, Yilong Wang

**Affiliations:** 1 Department of Neurology, Beijing Tiantan Hospital, Capital Medical University, Beijing 100070, China; guoliang_hu@163.com (G.H.); lizixiao2008@hotmail.com (Z.L.); 2 China National Clinical Research Center for Neurological Diseases, Beijing 100070, China; yuesongpan@ncrcnd.org.cn (Y.P.); wmxing1227@163.com (M.W.); mengxia45@163.com (X.M.); jiangyong@ncrcnd.org.cn (Y.J.); li_hao71@aliyun.com (H.L.); 3 Advanced Innovation Center for Human Brain Protection, Capital Medical University, Beijing 100070, China; 4 Beijing Key Laboratory of Translational Medicine for Cerebrovascular Disease, Beijing 100070, China; 5 Chinese Institute for Brain Research, Beijing 102206, China

**Keywords:** high-density lipoprotein cholesterol, lipids, acute ischemic stroke, diabetes mellitus, recurrent stroke, major adverse cardiovascular event, prospective study

## Abstract

A low high-density lipoprotein cholesterol (HDL-C) level is an identified risk factor for cardiovascular diseases. However, results on the association between HDL-C levels and adverse outcomes in diabetic status still remain limited and controversial. Herein, we evaluated the association between HDL-C levels and adverse outcomes among acute ischemic stroke (AIS) patients with diabetes mellitus. The cohort comprised 3824 AIS patients with diabetes mellitus (62.7 ± 10.5 years; 34.2% women) from the Third China National Stroke Registry (*n* = 15,166). Patients were classified into five groups by quintiles of HDL-C. The outcomes included recurrent stroke and major adverse cardiovascular events (MACEs) within 1 year. The relationship between HDL-C levels and the risk of adverse outcomes was analyzed by Cox proportional hazards models. Patients in the lowest quintile of HDL-C had a higher risk of recurrent stroke (hazard ratio (HR) 1.59, 95% confidence interval (CI), 1.12–2.25) and MACEs (HR 1.53, 95% CI, 1.09–2.15) during 1-year follow-up compared with those in the highest quintile of HDL-C. There were linear associations between HDL-C levels and the risks of both recurrent stroke and MACEs. Low HDL-C levels were associated with higher risks of recurrent stroke and MACEs within 1 year in AIS patients with diabetes mellitus.

## 1. Introduction

A low high-density lipoprotein cholesterol (HDL-C) level is an identified risk factor for cardiovascular diseases (CVDs) [1]. Previous studies have found a strong inverse association between HDL-C levels and worse outcomes in the general population [1,2,3]. Furthermore, experimental studies report that HDL-C plays an important role in preventing atherosclerosis due to its effect of reverse cholesterol transport (RCT), antioxidant, anti-inflammatory, anticoagulant, antiapoptotic, and vasodilatory activities, and role in endothelial function improvement [4,5,6]. However, increasing evidence implies that the levels and functionality of HDL-C may be reduced in the diabetic state [7,8], possibly owing to the liver’s lipid synthesis dysfunction and increased glycation/glycoxidation [9]. Previous findings regarding the association between HDL-C levels and adverse outcomes in the diabetic status remain limited and controversial [2,3,8,10,11,12,13]. A recent prospective community-based study showed a dose–response association between high HDL-C concentrations and high CVD risk in participants with diabetes mellitus [8], while other studies have reported opposing results [2,10,11,12,13]. In particular, the association between HDL-C and hemorrhagic stroke remains uncertain.

Diabetes mellitus is highly prevalent in acute ischemic stroke (AIS) patients in China [14,15]. The latest guidelines have classified AIS patients with diabetes mellitus as a special population considering their extremely high risk in the setting of secondary prevention [16,17,18]. Low levels of HDL-C as one of the major contributors for dyslipidemia in the Asian population have been found to be associated with an increased risk of major adverse cardiovascular events (MACEs) among AIS patients [12]. However, few studies have assessed whether low levels of HDL-C are also associated with an increased risk of adverse outcomes among AIS patients with diabetes mellitus.

The aim of the present study, therefore, was to evaluate the association between HDL-C levels and recurrent stroke and MACEs among AIS patients with diabetes mellitus based on data from the Third China National Stroke Registry (CNSR-III), a nationwide, large-scale, prospective cohort of patients with ischemic stroke in China.

## 2. Materials and Methods

### 2.1. Study Design

Our study was based on the CNSR-III, a nationwide, multicenter, prospective registry of acute ischemic cerebrovascular events in China. Details on the design and methodology of the CNSR-III project have been published elsewhere [19]. Briefly, the CNSR-III study included 201 participating hospitals from 22 provinces and 4 municipalities in China. A total of 15,166 patients aged ≥18 years with a diagnosis of AIS or transient ischemic attack (TIA), within 7 days from the onset of symptoms, were consecutively recruited from August 2015 to March 2018. The diagnosis of AIS was made according to the World Health Organization—defined criteria and confirmed by magnetic resonance imaging or computed tomography of the brain.

### 2.2. Study Population

In the current study, we focused on patients with ischemic stroke and diabetes mellitus in the pre-specified biomarker substudy of the CNSR-III. Of the 15,166 initially recruited patients, 3824 were analyzed after excluding patients with a diagnosis of TIA, those without diabetes mellitus, and those with missing HDL-C data. The flowchart of patient inclusion in the current study is shown in Supplementary Figure S1.

### 2.3. Baseline Data Collection

Baseline data including demographic characteristics, vascular risk factors, medical history, and medication use were collected by trained research coordinators through face-to-face interviews using a standardized operating protocol or were extracted from original medical records. Baseline medical history included hypertension, dyslipidemia, prior stroke, coronary heart disease, and atrial fibrillation. Diabetes mellitus was defined as having a previous or new diagnosis of diabetes mellitus, receiving anti-diabetic treatment, or having a fasting blood glucose level of ≥7.0 mmol/L (126 mg/dL) or hemoglobin A1c level of ≥6.5%. Dyslipidemia was defined as a serum triglyceride level of ≥2.3 mmol/L (200 mg/dL), low-density lipoprotein cholesterol (LDL-C) level of ≥4.1 mmol/L (160 mg/dL), HDL-C level of <1.0 mmol/L (40 mg/dL), receiving lipid-lowering drugs, or having a history of dyslipidemia [20]. Hypertension was defined as having a history of hypertension, receiving antihypertensive therapy, or having a systolic blood pressure ≥140 mmHg or diastolic blood pressure ≥90 mmHg at admission, or having hypertension listed as a discharge diagnosis in the medical records. Other clinical history of diseases, including history of dyslipidemia, prior stroke, coronary heart disease, and atrial fibrillation was defined based on the original medical records. Body mass index (BMI) was calculated as weight (kg) divided by height squared (m^2^). Severity of stroke on admission was measured by trained neurologists using the National Institutes of Health Stroke Scale (NIHSS) score [21]. Centralized etiological classification of ischemic stroke was performed according to the Trial of Org 10,172 in Acute Stroke Treatment (TOAST) criteria [22].

### 2.4. Blood Sample Collection and Laboratory Tests

Fasting blood samples from the CNSR-III patients were obtained within 24 h of hospital admission. Plasma specimens were extracted, aliquoted, and transported through cold chain to the core laboratory in Beijing Tiantan Hospital and stored at −80 °C until central blind testing. No freezing or thawing circle occurred before testing. The levels of HDL-C, LDL-C, and triglycerides were measured centrally.

### 2.5. Follow-Up and Outcomes

Patients were followed up at 6 and 12 months after stroke onset by trained research personnel via telephone interview based on a standardized interview protocol. Information on recurrent stroke and MACEs was collected. The definition of recurrent stroke of this study included ischemic stroke and hemorrhagic stroke. MACEs were defined as a combination of cardiovascular death, non-fatal ischemic stroke, non-fatal hemorrhagic stroke, and non-fatal myocardial infarction. Confirmation of stroke was sought from the attended hospital. Suspected recurrent stroke without hospitalization was judged by an independent endpoint judgment committee. Totally, 39 (1.02%) and 88 (2.30%) AIS patients with diabetes mellitus were lost to follow-up at 6 months and 1 year, respectively.

### 2.6. Statistical Analyses

Patients were divided into five groups by HDL-C quintiles in accordance with the method used in a previous study [23]. We described continuous variables as mean ± SD or median (interquartile range) and described categorical variables as proportions. Baseline characteristics stratified by HDL-C quintiles were compared by χ^2^ test for categorical variables and one-way ANOVA or Kruskal–Wallis test for continuous variables for difference between the five groups.

Cox proportional hazards models were used to calculate the hazard ratios (HRs) and 95% confidence intervals (CIs) for the risks of recurrent stroke and MACEs in the bottom quintile of HDL-C compared with the top quintile, and for a 1 mmol/L (39 mg/dL) increase in HDL-C levels. Tests for linear trends in HRs across the HDL-C quintiles were conducted using Cox regression models, with the median value of the corresponding quintile as the predictor. We performed two models in the multivariable analysis. We, in the first model, adjusted for age and sex. We adjusted for all confounding variables in the baseline in the second model, including age, sex, BMI, smoking, drinking, hypertension, history of dyslipidemia, history of stroke, history of atrial fibrillation, history of coronary heart disease, medication history of antiplatelet agents, medication history of lipid-lowering drugs, LDL-C level, triglycerides level, NIHSS score on admission, TOAST classification, use of antiplatelet agents at discharge, use of anticoagulant agents at discharge, and use of statins at discharge.

To visualize the relationship between HDL-C levels and recurrent stroke and MACEs, restricted cubic splines with five knots (at the 5th, 25th, 50th, 75th, and 95th centiles) were constructed. The reference point for the HDL-C level was the midpoint (0.89 mmol/L (34.36 mg/dL)) of the level of HDL-C, and the HR was adjusted for all potential confounders in Model 2. In addition, subgroup analyses, including age (<65 years/≥65 years), sex (male/female), smoking status (yes/no), drinking status (yes/no), hypertension (yes/no), NIHSS score at admission (<4/≥4), and ischemic stroke subtype (large-artery atherosclerosis/cardioembolism/small-artery occlusion/others) were performed by using clinically important characteristics.

All tests were two-tailed, and *p* < 0.05 was considered statistically significant. All analyses were conducted using SAS version 9.4 statistical software (SAS Institute, Inc. Cary, NC, USA).

## 3. Results

### 3.1. Baseline Characteristics of Patient Population

A total of 3824 patients with ischemic stroke and diabetes mellitus (2517 men and 1307 women) were included in the analysis. The mean (SD) age was 62.74 (10.54) years, and the median HDL-C level was 0.89 mmol/L (34.36 mg/dL; interquartile range, 0.74–1.06 mmol/L (28.57–40.93 mg/dL)). The proportion of patients with dyslipidemia was 75.84%, of which 98.41% received lipid-lowering medications. The baseline characteristics of patients stratified by the quintiles of HDL-C are shown in Table 1 and Supplementary Table S1. Compared with patients in the top quintile of HDL-C, those in the bottom quintile of HDL-C were more likely to be younger and male, and had a slightly higher BMI, higher levels of triglycerides, higher proportion of hypertension, dyslipidemia, and current smokers, while were more likely to have lower levels of LDL-C and to have a lower proportion of atrial fibrillation.

### 3.2. Associations between HDL-C Levels and Adverse Outcomes

The rate of adverse outcomes decreased across the quintiles of HDL-C (Table 2, Supplementary Table S2). From the lowest to the highest quintile, the rate of recurrent stroke decreased from 10.4% to 8.1% at 6 months (P for trend, 0.042) and from 12.3% to 9.6% at 12 months (P for trend, 0.023), and the rate of MACEs decreased from 11.0% to 8.3% at 6 months (P for trend, 0.028) and from 12.8% to 10.1% at 12 months (P for trend, 0.022). In an age- and sex-adjusted model, the HRs (95% CIs) for the lowest quintile of HDL-C were 1.43 (1.02–2.00) for recurrent stroke at 6 months, 1.41 (1.04–1.93) for recurrent stroke at 12 months, 1.46 (1.05–2.03) for MACEs at 6 months, and 1.39 (1.03–1.88) for MACEs at 12 months, compared with the highest quintile. After further adjusting the confounding variables in Model 2, patients in the lowest quintile of HDL-C levels still had higher risks of recurrent stroke and MACEs than those in the highest quintile; the HRs (95% CIs) for the lowest versus highest quintile of HDL-C were 1.56 (1.06–2.28) for recurrent stroke at 6 months, 1.59 (1.12–2.25) for recurrent stroke at 12 months, 1.59 (1.10–2.31) for MACEs at 6 months, and 1.53 (1.09–2.15) for MACEs at 12 months.

We then conducted a sensitivity analysis to evaluate the relationship between HDL-C levels and adverse outcomes. After excluding patients with stress hyperglycemia (*n* = 245), the inverse associations between HDL-C levels and the risks of recurrent stroke and MACEs still remained significant based on Model 2 (Supplementary Table S3). When we compared with patients with a normal HDL-C level (≥1 mmol/L (40 mg/dL)), we found patients with a low level of HDL-C (<1 mmol/L (40 mg/dL)) had a higher risk of adverse outcomes within 1 year (Supplementary Table S4).

**Figure 1:**
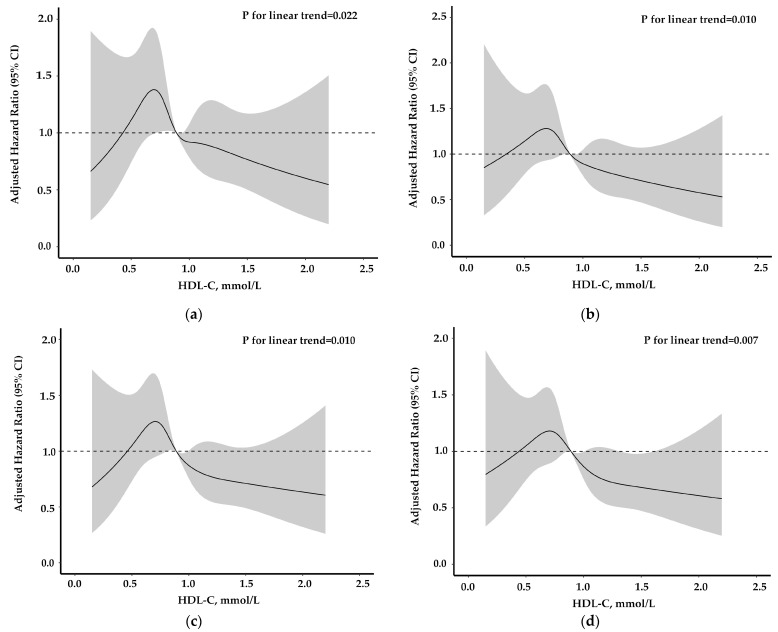
Adjusted hazard ratios of recurrent stroke and MACEs according to HDL-C levels. (**a**) Recurrent stroke within 6 months; (**b**) MACEs within 6 months; (**c**) Recurrent stroke within 12 months; (**d**) MACEs within 12 months. Hazard ratios and 95% CIs derived from restricted cubic spline regression, with 5 knots (at the 5th, 25th, 50th, 75th, and 95th centiles) for levels of HDL-C, adjusting for potential confounders in Model 2. The reference point for HDL-C is the midpoint (0.89 mmol/L (34.36 mg/dL)) of the levels of HDL-C. CI, confidence interval; HDL-C, high-density lipoprotein cholesterol; MACEs, major adverse cardiovascular events.

Furthermore, when we used HDL-C levels as a continuous variable, each 1 mmol/L (39 mg/dL) increase in HDL-C was associated with a 41% decreased risk of recurrent stroke at 6 months (HR, 0.59 (95% CI, 0.38–0.93)) and at 12 months (HR, 0.59 (95% CI, 0.40–0.88)), a 44% decreased risk of MACEs at 6 months (HR, 0.56 (95% CI, 0.36–0.87)), and a 41% decreased risk of MACEs at 12 months (HR, 0.59 (95% CI, 0.40–0.87)) among AIS patients with diabetes mellitus (Table 2). Multivariable adjusted restricted cubic spline analyses showed a linear association between HDL-C level and recurrent stroke and MACEs within 1 year (Figure 1).

In the subgroup analyses, there were no significant interactions of age, sex, smoking status, drinking status, hypertension, NIHSS score on admission, and ischemic stroke subtype with the risks of recurrent stroke and MACEs (all P for interaction, >0.05; Supplementary Table S5).

## 4. Discussion

In the present study, we found that HDL-C levels were inversely associated with the risks of recurrent stroke and MACEs within 1 year among ischemic stroke patients with diabetes mellitus, based on a large-scale, nationwide, multicenter, prospective registry study.

Low levels of HDL-C are regarded as the dominant components of dyslipidemia among Asian populations [24,25] and are highly prevalent in patients with diabetes mellitus, as over half of diabetic patients have low HDL-C levels [26]. The precise mechanism underlying the reduced HDL-C level in diabetes mellitus is unknown but may be a consequence of increased activities of cholesteryl ester transfer protein and endothelial lipase [9]. Several studies have indicated an inverse association between HDL-C levels and the risk of adverse outcomes among both the general population and high-risk patients [2,10,11,12,27]. A prospective analysis of 267,500 Chinese adults from six large cohorts suggested that each 1 mmol/L increase in HDL-C is associated with a 16% decreased risk of ischemic stroke (HR, 0.84, (95% CI, 0.78–0.90)) [10]. A recent study conducted among 512,891 adults from the China Kadoorie Biobank also showed that plasma concentrations of HDL-C are inversely associated with the risk of ischemic stroke (HR, 0.93, (95% CI, 0.89–0.97) per 0.3 mmol/L increase in HDL-C), independently of the LDL-C level and other conventional risk factors [2]. Moreover, the Louisiana Experiment Assessing Diabetes outcomes (LEAD) cohort study also found consistent inverse associations between HDL-C levels and the risks of total (HR, 0.89, (95% CI, 0.86–0.91) per 0.39 mmol/L (15 mg/dL) increase in HDL-C) and ischemic stroke (HR, 0.89, (95% CI, 0.86–0.92) per 0.39 mmol/L (15 mg/dL) increase in HDL-C) among patients with diabetes mellitus [11]. Consistent with previous studies, we also found an inverse association between HDL-C levels and the risks of recurrent total and ischemic stroke among AIS patients with diabetes mellitus. Additionally, the inverse association still remained significant after excluding patients with stress hyperglycemia in our study. Of note, the degree of risk reduction for adverse outcomes in our study was relatively larger than that reported in previous study. The disparities in results may be mainly attributed to differences in HDL-C levels. The levels of HDL-C, on average, were lower in our study than in previous study (0.92 ± 0.28 mmol/L vs. 1.43 ± 0.40 mmol/L). Therefore, the degree of risk reduction caused by each unit increase in HDL-C in our study was greater than that reported in previous study. Additionally, differences between study populations may also contribute to the interstudy discrepancy in the HRs of HDL-C levels for adverse outcomes. Most previous studies focused on the general population without CVD, while the participants in our study were diabetic AIS patients with a higher proportion of multiple risk factors (e.g., hypertension and higher BMI) and were, on average, substantially older.

For hemorrhagic stroke, its relationship with HDL-C levels remains limited and controversial. In our study, we found no relationship between HDL-C levels and recurrent hemorrhagic stroke. Most previous studies reported findings similar to ours [2,3], whereas other studies showed an inverse association between HDL-C levels and hemorrhagic stroke [10,11]. In a retrospective cohort study of 67,544 participants from LEAD study, HDL-C level is inversely associated with the risk of hemorrhagic stroke (HR, 0.83, (95% CI, 0.73–0.95) per 0.39 mmol/L (15 mg/dL) increase in HDL-C) among patients with diabetes mellitus; however, there was no further decrease in the risk of hemorrhagic stroke among the group with very high HDL-C levels [11]. Furthermore, a large prospective cohort study showed that compared with subjects with a HDL-C level of 1.30 to 1.55 mmol/L (50 to 59.9 mg/dL), the risk of hemorrhagic stroke is increased in those with HDL-C levels lower than 1.30 mmol/L (50 mg/dL) (<1.04 mmol/L (40 mg/dL): HR, 1.28, (95% CI, 1.10–1.49); 1.04 to 1.29 mmol/L (40 to 49.9 mg/dL): HR, 1.17, (95% CI, 1.03–1.33)) [10]. These interstudy differences may mainly result from the variation in the incidence of hemorrhagic stroke events among different study populations. Further studies with examination of levels of HDL-C and the risk of hemorrhagic stroke are needed, especially among the diabetic status.

Previous studies have showed inconsistent results regarding the association between HDL-C levels and MACEs [8,28,29,30]. Our study showed a significantly higher risk of MACEs in lower HDL-C concentration groups than in higher HDL-C concentration groups. In line with our findings, a hospital-based study reported that a low HDL-C level at admission is a significant independent predictor of an adverse composite endpoint, independent of the effects of LDL-C, among patients with atherosclerotic ischemic stroke (HR, 1.41, (95% CI, 1.02–1.95)) [12]. Similarly, a subanalysis of the Heart Institute of Japan-PRoper level of lipid lOwering with Pitavastatin and Ezetimibe in acute coRonary syndrome (HIJ-PROPER) study also showed that lower levels of HDL-C are associated with increased risk of cardiovascular events (HR, 1.47, (95% CI, 1.12–1.94)) in acute coronary syndrome patients receiving contemporary lipid-lowering therapy [31]. Furthermore, the Japan Cholesterol and Diabetes Mellitus study also showed an inverse association between HDL-C levels and the risk of CVD among patients with diabetes mellitus, especially in diabetic elderly individuals [32]. Together, these studies were in favor of our results. However, in the most recent study conducted among participants from the Kailuan study, HDL-C is positively associated with the risk of CVD in individuals with diabetes mellitus, but low HDL-C levels fail to predict future CVD risk in the presence of diabetes mellitus [8]. That is, high levels of HDL-C are associated with a high risk of CVD (HR, 1.62, (95% CI, 1.19–2.20)) compared with intermediate levels of HDL-C in diabetic individuals [8]. In addition, these results were further confirmed by using a decile-based analysis. There are several possible mechanisms underlying the changed relationship between HDL-C levels and adverse outcomes in the diabetic population compared with the general population. Hyperglycemia may modify HDL-C function, making it a poorer predictor in diabetic individuals. Increased glycation/glycoxidation of HDL-C impairs its RCT ability and diminishes its antiatherogenic capacity [7]. Additionally, the usage of traditional Chinese medicines might potentially interfere with lipid metabolism and function. However, our hospital-based study still found an inverse association between HDL-C levels and MACEs. The absence of a consistent relationship between HDL-C levels and the risk of MACEs may be due to differences in the sex composition of study populations, inconsistent definitions of diabetes mellitus and composite outcomes, and differences in confounding factors adjusted in multivariable analysis models.

Several limitations should be noted here. First, the levels of HDL-C were examined only at baseline but not at follow-up. Dynamic changes in HDL-C concentrations may influence the long-term prognosis. Second, limited information regarding types of diabetes and HDL-C functionality were collected in the present study. More prospective studies with data on diabetes type and the particle size and subclass concentration of HDL-C are warranted. Third, although our analyses adjusted for some confounding factors, the findings may have been affected by other unmeasured or residual factors. Fourth, dyslipidemia rarely occurred in isolation in our study population. Further studies that consider isolated dyslipidemia are warranted. Finally, as all patients in this study were Chinese, it is unknown whether the results of this study can be extrapolated to patients outside of East Asia.

## 5. Conclusions

In AIS patients with diabetes mellitus, low levels of HDL-C were associated with increased risks of recurrent stroke and MACEs within 1 year. Our study provides further reliable evidence for an inverse association between HDL-C levels and the risks of adverse outcomes in AIS patients with diabetes mellitus, especially among the Asian population.

## Figures and Tables

**Table 1:** Characteristics of patients included according to HDL-C quintiles.

Characteristics	Overall	HDL-C Quintiles					*p* Value
		Q1, <0.71 mmol/L	Q2, 0.71–0.83 mmol/L	Q3, 0.83–0.95 mmol/L	Q4, 0.95–1.11 mmol/L	Q5, ≥1.11 mmol/L	
Patients, *n*	3824	757	712	792	780	783	
Age, mean (SD), years	62.74 ± 10.54	61.89 ± 10.83	61.39 ± 10.59	62.38 ± 10.51	62.91 ± 10.30	65.00 ± 10.13	<0.001
Women, *n* (%)	1307 (34.18)	188 (24.83)	185 (25.98)	256 (32.32)	278 (35.64)	400 (51.09)	<0.001
BMI, mean (SD)	25.16 ± 3.31	25.53 ± 3.40	25.33 ± 3.23	25.17 ± 3.17	25.01 ± 3.10	24.77 ± 3.57	<0.001
LDL-C, median (IQR), mmol/L	2.35 (1.70–3.04)	2.05 (1.42–2.69)	2.22 (1.64–2.88)	2.30 (1.70–2.94)	2.38 (1.77–3.04)	2.73 (2.02–3.57)	<0.001
TG, median (IQR), mmol/L	1.50 (1.14–2.11)	1.79 (1.32–2.54)	1.64 (1.26–2.32)	1.53 (1.18–2.09)	1.40 (1.08–1.88)	1.24 (0.98–1.71)	<0.001
Medical history, *n* (%)							
Hypertension	3502 (91.58)	704 (93.00)	637 (89.47)	732 (92.42)	702 (90.00)	727 (92.85)	0.027
Dyslipidemia	2900 (75.84)	757 (100.00)	712 (100.00)	792 (100.00)	417 (53.46)	222 (28.35)	<0.001
Current smoking	1143 (29.89)	266 (35.14)	256 (35.96)	243 (30.68)	220 (28.21)	158 (20.18)	<0.001
Drinking	517 (13.52)	105 (13.87)	98 (13.76)	108 (13.64)	104 (13.33)	102 (13.03)	0.989
Stroke	941 (24.61)	204 (26.95)	171 (24.02)	203 (25.63)	182 (23.33)	181 (23.12)	0.350
Coronary heart disease	503 (13.15)	112 (14.80)	75 (10.53)	107 (13.51)	113 (14.49)	96 (12.26)	0.095
Atrial fibrillation	230 (6.01)	44 (5.81)	32 (4.49)	34 (4.29)	58 (7.44)	62 (7.92)	0.005
Medication history, *n* (%)							
Antiplatelet agents	764 (19.98)	173 (22.85)	146 (20.51)	166 (20.96)	134 (17.18)	145 (18.52)	0.052
Lipid-lowering agents	489 (12.79)	107 (14.13)	90 (12.64)	107 (13.51)	87 (11.15)	98 (12.52)	0.473
NIHSS on admission, median (IQR)	4.00 (2.00–6.00)	4.00 (2.00–6.00)	4.00 (2.00–6.00)	4.00 (2.00–6.00)	4.00 (2.00–6.00)	4.00 (2.00–6.00)	0.595
Ischemic stroke subtype, *n* (%)							0.023
Large-artery atherosclerosis	1059 (27.69)	217 (28.67)	219 (30.76)	234 (29.55)	195 (25.00)	194 (24.78)	
Cardiac embolism	212 (5.54)	37 (4.89)	29 (4.07)	36 (4.55)	54 (6.92)	56 (7.15)	
Small-vessel occlusion	863 (22.57)	166 (21.93)	169 (23.74)	185 (23.36)	175 (22.44)	168 (21.46)	
Other	1690 (44.19)	337 (44.52)	295 (41.43)	337 (42.55)	356 (45.64)	365 (46.62)	
Medication at discharge, *n* (%)							
Antiplatelet agents	3529 (92.55)	699 (92.83)	658 (92.68)	740 (93.43)	723 (92.93)	709 (90.90)	0.373
Anticoagulant agents	84 (2.20)	16 (2.12)	14 (1.97)	15 (1.89)	23 (2.96)	16 (2.05)	0.613
Statins	3540 (99.72)	696 (99.57)	657 (99.70)	736 (99.73)	729 (99.73)	722 (99.86)	0.896

BMI, body mass index; HDL-C, high-density lipoprotein cholesterol; IQR, interquartile range; LDL-C, low-density lipoprotein cholesterol; NIHSS, National Institutes of Health Stroke Scale; TG, triglyceride.

**Table 2:** Hazard ratios (95% CIs) for risk of adverse outcomes according to quintiles of HDL-C.

Outcome	HDL-C Quintiles, mmol/L					P for Trend	Each 1-mmol/L Increase in HDL-C
	Q1, <0.71 mmol/L	Q2, 0.71–0.83 mmol/L	Q3, 0.83–0.95 mmol/L	Q4, 0.95–1.11 mmol/L	Q5, ≥1.11 mmol/L		
6 months							
Recurrent stroke							
Events, *n* (%)	79 (10.44)	72 (10.11)	66 (8.33)	64 (8.21)	63 (8.05)		
Adjusted HR *	1.43 (1.02–2.00)	1.40 (0.99–1.97)	1.11 (0.79–1.58)	1.08 (0.76–1.53)	1.00	0.015	0.68 (0.46–1.01)
Adjusted HR †	1.56 (1.06–2.28)	1.50 (1.03–2.20)	1.20 (0.82–1.75)	1.09 (0.74–1.59)	1.00	0.007	0.59 (0.38–0.93)
MACEs							
Events, *n* (%)	83 (10.96)	76 (10.67)	72 (9.09)	67 (8.59)	65 (8.30)		
Adjusted HR *	1.46 (1.05–2.03)	1.43 (1.02–2.00)	1.18 (0.84–1.65)	1.10 (0.78–1.54)	1.00	0.009	0.66 (0.45–0.96)
Adjusted HR †	1.59 (1.10–2.31)	1.54 (1.07–2.24)	1.27 (0.88–1.84)	1.08 (0.74–1.57)	1.00	0.003	0.56 (0.36–0.87)
12 months							
Recurrent stroke							
Events, *n* (%)	93 (12.29)	89 (12.50)	85 (10.73)	76 (9.74)	75 (9.58)		
Adjusted HR *	1.41 (1.04–1.93)	1.45 (1.06–1.98)	1.20 (0.88–1.64)	1.07 (0.78–1.48)	1.00	0.007	0.70 (0.49–1.00)
Adjusted HR †	1.59 (1.12–2.25)	1.57 (1.11–2.21)	1.32 (0.94–1.86)	1.11 (0.78–1.57)	1.00	0.002	0.59 (0.40–0.88)
MACEs							
Events, *n* (%)	97 (12.81)	94 (13.20)	93 (11.74)	80 (10.26)	79 (10.09)		
Adjusted HR *	1.39 (1.03–1.88)	1.44 (1.06–1.96)	1.24 (0.92–1.68)	1.07 (0.78–1.46)	1.00	0.007	0.70 (0.49–0.98)
Adjusted HR †	1.53 (1.09–2.15)	1.54 (1.11–2.16)	1.36 (0.98–1.88)	1.07 (0.76–1.50)	1.00	0.002	0.59 (0.40–0.87)

HDL-C, high-density lipoprotein cholesterol; HR, hazard ratio; MACEs, major adverse cardiovascular events. * Model 1: age, sex. † Model 2: age, sex, body mass index, smoking, drinking, hypertension, history of dyslipidemia, history of stroke, history of atrial fibrillation, history of coronary heart disease, medication history of antiplatelet agents, medication history of lipid-lowering drugs, low-density lipoprotein cholesterol, triglycerides, National Institutes of Health Stroke Scale on admission, TOAST, use of antiplatelet agents at discharge, use of anticoagulant agents at discharge, and use of statins at discharge.

## Data Availability

The data will be available to researchers on reasonable request for purpose of reproducing results or replicating the procedure from the corresponding author.

## Supplementary Materials

The following are available online at https://www.mdpi.com/article/10.3390/biomedicines9121947/s1, Table S1: Characteristics of Patients Included According to HDL-C Quintiles, Table S2: Hazard Ratios (95% CIs) for Risk of Adverse Outcomes According to Quintiles of HDL-C in Patients Included in This Study, Table S3: Sensitivity Analysis for the Association between HDL-C and Adverse Outcomes, Table S4: Multivariable Analysis of Association between Low Levels of HDL-C and Adverse Outcomes within 1 year Compared with Normal Levels of HDL-C, Table S5.1: Subgroup Analysis for the Association between HDL-C and Recurrent Stroke at 6 Months, Table S5.2: Subgroup Analysis for the Association between HDL-C and MACEs at 6 Months, Table S5.3: Subgroup Analysis for the Association between HDL-C and Recurrent Stroke at 12 Months, Table S5.4: Subgroup Analysis for the Association between HDL-C and MACEs at 12 Months, Figure S1: Flow Chart of the Study Population.

## References

[1] Prospective Studies Collaboration (2007). Blood cholesterol and vascular mortality by age, sex, and blood pressure: A meta-analysis of individual data from 61 prospective studies with 55,000 vascular deaths. Lancet.

[2] Sun L., Clarke R., Bennett D., Guo Y., Walters R.G., Hill M., Parish S., Millwood I.Y., Bian Z., Chen Y. (2019). Causal associations of blood lipids with risk of ischemic stroke and intracerebral hemorrhage in Chinese adults. Nat. Med..

[3] Zhang Y., Tuomilehto J., Jousilahti P., Wang Y., Antikainen R., Hu G. (2012). Total and high-density lipoprotein cholesterol and stroke risk. Stroke.

[4] Navab M., Hama S.Y., Cooke C.J., Anantharamaiah G.M., Chaddha M., Jin L., Subbanagounder G., Faull K.F., Reddy S.T., Miller N.E. (2000). Normal high density lipoprotein inhibits three steps in the formation of mildly oxidized low density lipoprotein: Step 1. J. Lipid Res..

[5] Navab M., Hama S.Y., Anantharamaiah G.M., Hassan K., Hough G.P., Watson A.D., Reddy S.T., Sevanian A., Fonarow G.C., Fogelman A.M. (2000). Normal high density lipoprotein inhibits three steps in the formation of mildly oxidized low density lipoprotein: Steps 2 and 3. J. Lipid Res..

[6] Lee C.K., Liao C.W., Meng S.W., Wu W.K., Chiang J.Y., Wu M.S. (2021). Lipids and Lipoproteins in Health and Disease: Focus on Targeting Atherosclerosis. Biomedicines.

[7] Hoang A., Murphy A.J., Coughlan M.T., Thomas M.C., Forbes J.M., O’Brien R., Cooper M.E., Chin-Dusting J.P., Sviridov D. (2007). Advanced glycation of apolipoprotein A-I impairs its anti-atherogenic properties. Diabetologia.

[8] Wu Z., Huang Z., Lichtenstein A.H., Jin C., Chen S., Wu S., Gao X. (2021). Different associations between HDL cholesterol and cardiovascular diseases in people with diabetes mellitus and people without diabetes mellitus: A prospective community-based study. Am. J. Clin. Nutr..

[9] Barter P.J. (2013). High density lipoprotein: A therapeutic target in type 2 diabetes. Endocrinol. Metab..

[10] Gu X., Li Y., Chen S., Yang X., Liu F., Li Y., Li J., Cao J., Liu X., Chen J. (2019). Association of Lipids With Ischemic and Hemorrhagic Stroke. Stroke.

[11] Shen Y., Shi L., Nauman E., Katzmarzyk P.T., Price-Haywood E.G., Bazzano A.N., Nigam S., Hu G. (2019). Inverse Association Between HDL (High-Density Lipoprotein) Cholesterol and Stroke Risk Among Patients with Type 2 Diabetes Mellitus. Stroke.

[12] Yeh P.S., Yang C.M., Lin S.H., Wang W.M., Chen P.S., Chao T.H., Lin H.J., Lin K.C., Chang C.Y., Cheng T.J. (2013). Low levels of high-density lipoprotein cholesterol in patients with atherosclerotic stroke: A prospective cohort study. Atherosclerosis.

[13] Luo Y., Li J., Zhang J., Xu Y. (2014). Low HDL cholesterol is correlated to the acute ischemic stroke with diabetes mellitus. Lipids Health Dis..

[14] Jia Q., Zheng H., Zhao X., Wang C., Liu G., Wang Y., Liu L., Li H., Zhong L., Wang Y. (2012). Abnormal glucose regulation in patients with acute stroke across China: Prevalence and baseline patient characteristics. Stroke.

[15] Wang Y.J., Li Z.X., Gu H.Q., Zhai Y., Jiang Y., Zhao X.Q., Wang Y.L., Yang X., Wang C.J., Meng X. (2020). China Stroke Statistics 2019: A Report From the National Center for Healthcare Quality Management in Neurological Diseases, China National Clinical Research Center for Neurological Diseases, the Chinese Stroke Association, National Center for Chronic and Non-communicable Disease Control and Prevention, Chinese Center for Disease Control and Prevention and Institute for Global Neuroscience and Stroke Collaborations. Stroke Vasc. Neurol..

[16] Garber A.J., Handelsman Y., Grunberger G., Einhorn D., Abrahamson M.J., Barzilay J.I., Blonde L., Bush M.A., DeFronzo R.A., Garber J.R. (2020). Consensus Statement by the American Association of Clinical Endocrinologists and American College of Endocrinology on the Comprehensive Type 2 Diabetes Management Algorithm—2020 Executive Summary. Endocr. Pract..

[17] Powers W.J., Rabinstein A.A., Ackerson T., Adeoye O.M., Bambakidis N.C., Becker K., Biller J., Brown M., Demaerschalk B.M., Hoh B. (2019). Guidelines for the Early Management of Patients With Acute Ischemic Stroke: 2019 Update to the 2018 Guidelines for the Early Management of Acute Ischemic Stroke: A Guideline for Healthcare Professionals From the American Heart Association/American Stroke Association. Stroke.

[18] Wang Y., Han S., Qin H., Zheng H., Jiang B., Cao Y., Gao Y., Guan L., Jia Q., Jiang Y. (2020). Chinese Stroke Association guidelines for clinical management of cerebrovascular disorders: Executive summary and 2019 update of the management of high-risk population. Stroke Vasc. Neurol..

[19] Wang Y., Jing J., Meng X., Pan Y., Wang Y., Zhao X., Lin J., Li W., Jiang Y., Li Z. (2019). The Third China National Stroke Registry (CNSR-III) for patients with acute ischaemic stroke or transient ischaemic attack: Design, rationale and baseline patient characteristics. Stroke Vasc. Neurol..

[20] Joint Committee for Guideline Revision National Expert Committee on Cardiovascular Diseases (2018). 2016 Chinese guidelines for the management of dyslipidemia in adults. J. Geriatr. Cardiol..

[21] Brott T., Adams H.P., Olinger C.P., Marler J.R., Barsan W.G., Biller J., Spilker J., Holleran R., Eberle R., Hertzberg V. (1989). Measurements of acute cerebral infarction: A clinical examination scale. Stroke.

[22] Adams H.P., Bendixen B.H., Kappelle L.J., Biller J., Love B.B., Gordon D.L., Marsh E.E. (1993). Classification of subtype of acute ischemic stroke. Definitions for use in a multicenter clinical trial. TOAST. Trial of Org 10172 in Acute Stroke Treatment. Stroke.

[23] Ohm J., Hjemdahl P., Skoglund P.H., Discacciati A., Sundstrom J., Hambraeus K., Jernberg T., Svensson P. (2019). Lipid levels achieved after a first myocardial infarction and the prediction of recurrent atherosclerotic cardiovascular disease. Int. J. Cardiol..

[24] Huxley R.R., Barzi F., Lam T.H., Czernichow S., Fang X., Welborn T., Shaw J., Ueshima H., Zimmet P., Jee S.H. (2011). Isolated low levels of high-density lipoprotein cholesterol are associated with an increased risk of coronary heart disease: An individual participant data meta-analysis of 23 studies in the Asia-Pacific region. Circulation.

[25] Yang W., Xiao J., Yang Z., Ji L., Jia W., Weng J., Lu J., Shan Z., Liu J., Tian H. (2012). Serum lipids and lipoproteins in Chinese men and women. Circulation.

[26] Chatterji P., Joo H., Lahiri K. (2012). Racial/ethnic- and education-related disparities in the control of risk factors for cardiovascular disease among individuals with diabetes. Diabetes Care.

[27] Zhang Y., Li J., Liu C., Yu H., Chen C., Bi C., Fang C., Ma H., Li A., Dong Q. (2021). High-Density Lipoprotein Cholesterol and the Risk of First Ischemic Stroke in a Chinese Hypertensive Population. Clin. Interv. Aging.

[28] Wang X., Pei J., Zheng K., Hu X. (2021). High-density lipoprotein cholesterol levels are associated with major adverse cardiovascular events in male but not female patients with hypertension. Clin. Cardiol..

[29] Kaze A.D., Santhanam P., Musani S.K., Ahima R., Echouffo-Tcheugui J.B. (2021). Metabolic Dyslipidemia and Cardiovascular Outcomes in Type 2 Diabetes Mellitus: Findings From the Look AHEAD Study. J. Am. Heart Assoc..

[30] Han B.H., Han K., Yoon K.H., Kim M.K., Lee S.H. (2020). Impact of Mean and Variability of High-Density Lipoprotein-Cholesterol on the Risk of Myocardial Infarction, Stroke, and Mortality in the General Population. J. Am. Heart Assoc..

[31] Nakazawa M., Arashi H., Yamaguchi J., Ogawa H., Hagiwara N. (2020). Lower levels of high-density lipoprotein cholesterol are associated with increased cardiovascular events in patients with acute coronary syndrome. Atherosclerosis.

[32] Hayashi T., Kawashima S., Itoh H., Yamada N., Sone H., Watanabe H., Hattori Y., Ohrui T., Yokote K., Nomura H. (2009). Low HDL cholesterol is associated with the risk of stroke in elderly diabetic individuals: Changes in the risk for atherosclerotic diseases at various ages. Diabetes Care.

