# Supplementary material for: Inverse Association between High-Density Lipoprotein Cholesterol and Adverse Outcomes among Acute Ischemic Stroke Patients with Diabetes Mellitus
Source: Biomedicines. 2021 Dec 20;9(12):1947. doi: 10.3390/biomedicines9121947 (PMC8698640; doi:10.3390/biomedicines9121947)
Supplement: Supplementary file 1 [file biomedicines-09-01947-s001.zip › biomedicines-1508904-supplementary.pdf]

Supplementary Materials

Inverse Association Between High-Density Lipoprotein Cholesterol and Adverse Outcomes Among Acute Ischemic Stroke Patients with Diabetes Mellitus

Guoliang Hu [1,2,3,4], Yuesong Pan [2], Mengxing Wang [2], Xia Meng [2], Yong Jiang [2], Zixiao Li [1,2,5], Hao Li [2], Yongjun Wang [1,2,3,4,*] and Yilong Wang [1,2,3,4,5,*]

1 Department of Neurology, Beijing Tiantan Hospital, Capital Medical University, Beijing, China; guoliang_hu@163.com (G.L.H.); lizixiao2008@hotmail.com (Z.X.L.)

2 China National Clinical Research Center for Neurological Diseases, Beijing, China; yuesongpan@ncrcnd.org.cn (Y.S.P.); wmxing1227@163.com (M.X.W.); mengxia45@163.com (X.M.); jiangyong@ncrcnd.org.cn (Y.J.); li_hao71@aliyun.com (H.L.)

3 Advanced Innovation Center for Human Brain Protection, Capital Medical University, Beijing, China

4 Beijing Key Laboratory of Translational Medicine for Cerebrovascular Disease, Beijing, China

5 Chinese Institute for Brain Research, Beijing, China

* Correspondence: yongjunwang@ncrcnd.org.cn (Y.J.W.); yilong528@aliyun.com (Y.L.W.)

Supplementary Tables

Supplementary Table S1 Characteristics of Patients Included According to HDL-C Quintiles

Characteristics	Overall	HDL-C quintiles					P value
		Q1, <0.71 mmol/L	Q2, 0.71-0.83 mmol/L	Q3, 0.83-0.95 mmol/L	Q4, 0.95-1.11 mmol/L	Q5, ≥1.11 mmol/L	
**Patients, n**	3,824	757	712	792	780	783	
**SBP, mean (SD)**	152.53±22.18	151.65±21.87	150.05±22.10	153.00±20.92	152.72±22.32	154.99±23.39	<0.001
**DBP, mean (SD)**	87.59±13.00	87.52±12.65	87.41±12.88	87.68±13.06	87.47±12.72	87.84±13.66	0.969
**Medical history, n (%)**							
**History of dyslipidemia**	409 (10.70)	92 (12.15)	78 (10.96)	80 (10.10)	85 (10.90)	74 (9.45)	0.504
**Heart failure**	27 (0.71)	10 (1.32)	3 (0.42)	4 (0.51)	3 (0.38)	7 (0.89)	0.141
**Medication history, n (%)**							
**Antihypertensive agents**	1,944 (50.84)	394 (52.05)	335 (47.05)	418 (52.78)	383 (49.10)	414 (52.87)	0.093
**NIHSS on admission, n (%)**							0.634
**0-5**	2,677 (70.01)	529 (69.88)	504 (70.79)	563 (71.09)	543 (69.62)	538 (68.71)	
**6-15**	1,064 (27.82)	205 (27.08)	195 (27.39)	217 (27.40)	218 (27.95)	229 (29.25)	
**>=16**	83 (2.17)	23 (3.04)	13 (1.83)	12 (1.52)	19 (2.44)	16 (2.04)	
**Medication at discharge, n (%)**							
**Antihypertensive agents**	2,083 (54.63)	414 (54.98)	370 (52.11)	433 (54.67)	423 (54.37)	443 (56.79)	0.501

DBP, diastolic blood pressure; HDL-C, high-density lipoprotein cholesterol; IQR, interquartile range; NIHSS, National Institutes of Health Stroke Scale; SBP, systolic blood pressure.

Supplementary Table S2 Hazard Ratios (95% CIs) for Risk of Adverse Outcomes According to Quintiles of HDL-C in Patients Included in This Study

Outcome	HDL-C quintiles, mmol/L					P for trend	Each 1-mmol/L increase in HDL-C
	Q1, <0.71 mmol/L	Q2, 0.71-0.83 mmol/L	Q3, 0.83-0.95 mmol/L	Q4, 0.95-1.11 mmol/L	Q5, ≥1.11 mmol/L		
**6 months**							
**Recurrent stroke**							
**Recurrent ischemic stroke**							
Events, n (%)	75 (9.91)	66 (9.27)	64 (8.08)	61 (7.82)	60 (7.66)		
Incidence, per 1000 person-years	220.64	206.13	178.22	171.55	167.77		
Adjusted HR*	1.44 (1.02-2.03)	1.36 (0.95-1.94)	1.14 (0.80-1.63)	1.09 (0.76-1.56)	1.00	0.020	0.66 (0.44-0.98)
Adjusted HR†	1.54 (1.05-2.28)	1.46 (0.99-2.15)	1.20 (0.82-1.76)	1.06 (0.72-1.57)	1.00	0.010	0.56 (0.36-0.89)
**Recurrent hemorrhagic stroke**							
Events, n (%)	5 (0.66)	7 (0.98)	4 (0.51)	4 (0.51)	4 (0.51)		
Incidence, per 1000 person-years	13.76	20.37	10.50	10.64	10.64		
Adjusted HR*	1.17 (0.31-4.45)	1.76 (0.51-6.14)	0.92 (0.23-3.72)	0.95 (0.24-3.81)	1.00	0.557	1.20 (0.29-4.86)
Adjusted HR†	1.71 (0.33-8.74)	1.71 (0.33-8.92)	1.57 (0.32-7.64)	1.75 (0.35-8.66)	1.00	0.491	1.06 (0.24-4.71)
**MACEs**							
**Cardiovascular death**							
Events, n (%)	6 (0.79)	6 (0.84)	9 (1.14)	7 (0.90)	13 (1.66)		
Incidence, per 1000 person-years	16.43	17.41	23.59	18.58	34.48		
Adjusted HR*	0.52 (0.20-1.39)	0.57 (0.21-1.52)	0.74 (0.31-1.75)	0.58 (0.23-1.45)	1.00	0.179	2.19 (0.92-5.18)
Adjusted HR†	0.45 (0.11-1.87)	0.53 (0.13-2.20)	1.25 (0.44-3.53)	0.75 (0.24-2.39)	1.00	0.346	1.92 (0.57-6.45)
**Non-fatal stroke**							
Events, n (%)	40 (5.28)	30 (4.21)	22 (2.78)	25 (3.21)	28 (3.58)		
Incidence, per 1000 person-years	112.57	88.84	58.49	67.49	75.67		
Adjusted HR*	1.58 (0.97-2.59)	1.25 (0.74-2.11)	0.81 (0.46-1.42)	0.93 (0.54-1.59)	1.00	0.045	0.73 (0.40-1.33)

Outcome	HDL-C quintiles, mmol/L					P for trend	Each 1-mmol/L increase in HDL-C
	Q1, <0.71 mmol/L	Q2, 0.71-0.83 mmol/L	Q3, 0.83-0.95 mmol/L	Q4, 0.95-1.11 mmol/L	Q5, ≥1.11 mmol/L		
Adjusted HR†	1.60 (0.94-2.72)	1.27 (0.73-2.2)	0.80 (0.45-1.44)	0.90 (0.51-1.57)	1.00	0.055	0.72 (0.38-1.38)
**Non-fatal MI**							
Events, n (%)	3 (0.40)	1 (0.14)	4 (0.51)	1 (0.13)	0 (0.00)		
Incidence, per 1000 person-years	8.22	2.90	10.50	2.66	0		
Adjusted HR*	-	-	-	-	-	-	0.06 (0.01-0.89)
Adjusted HR†	-	-	-	-	-	-	0.07 (0.004-1.10)
**12 months**							
**Recurrent stroke**							
**Recurrent ischemic stroke**							
Events, n (%)	86 (11.36)	82 (11.52)	83 (10.48)	71 (9.10)	69 (8.81)		
Incidence, per 1000 person-years	129.26	131.16	117.71	101.68	98.40		
Adjusted HR*	1.44 (1.04-1.99)	1.47 (1.06-2.04)	1.29 (0.94-1.78)	1.10 (0.79-1.54)	1.00	0.007	0.66 (0.45-0.95)
Adjusted HR†	1.61 (1.12-2.31)	1.60 (1.12-2.29)	1.41 (1.00-2.00)	1.11 (0.77-1.59)	1.00	0.002	0.54 (0.35-0.81)
**Recurrent hemorrhagic stroke**							
Events, n (%)	8 (1.06)	9 (1.26)	5 (0.63)	6 (0.77)	7 (0.89)		
Incidence, per 1000 person-years	11.10	13.26	6.61	8.05	9.40		
Adjusted HR*	1.01 (0.36-2.83)	1.22 (0.45-3.33)	0.63 (0.20-1.99)	0.78 (0.26-2.33)	1.00	0.763	1.47 (0.48-4.47)
Adjusted HR†	1.30 (0.40-4.21)	1.15 (0.35-3.82)	0.95 (0.28-3.25)	1.12 (0.34-3.64)	1.00	0.627	1.32 (0.40-4.34)
**MACEs**							
**Cardiovascular death**							
Events, n (%)	8 (1.06)	7 (0.98)	13 (1.64)	8 (1.03)	19 (2.43)		
Incidence, per 1000 person-years	11.02	10.26	17.15	10.70	25.42		

Outcome	HDL-C quintiles, mmol/L					P for trend	Each 1-mmol/L increase in HDL-C
	Q1, <0.71 mmol/L	Q2, 0.71-0.83 mmol/L	Q3, 0.83-0.95 mmol/L	Q4, 0.95-1.11 mmol/L	Q5, ≥1.11 mmol/L		
Adjusted HR*	0.43 (0.19-1.00)	0.41 (0.17-0.99)	0.68 (0.33-1.38)	0.42 (0.18-0.97)	1.00	0.034	2.50 (1.22-5.12)
Adjusted HR†	0.40 (0.14-1.18)	0.33 (0.10-1.06)	0.89 (0.40-2.02)	0.43 (0.16-1.15)	1.00	0.086	2.30 (0.88-5.98)
**Non-fatal stroke**							
Events, n (%)	53 (7.00)	49 (6.88)	41 (5.18)	37 (4.74)	37 (4.73)		
Incidence, per 1000 person-years	76.25	74.57	55.56	50.92	51.07		
Adjusted HR*	1.59 (1.04-2.44)	1.56 (1.01-2.41)	1.15 (0.73-1.79)	1.04 (0.66-1.64)	1.00	0.009	0.69 (0.42-1.14)
Adjusted HR†	1.74 (1.10-2.76)	1.64 (1.04-2.60)	1.22 (0.77-1.95)	1.01 (0.63-1.64)	1.00	0.004	0.60 (0.35-1.04)
**Non-fatal MI**							
Events, n (%)	3 (0.40)	2 (0.28)	5 (0.63)	2 (0.26)	0 (0.00)		
Incidence, per 1000 person-years	4.14	2.93	6.62	2.68	0		
Adjusted HR*	-	-	-	-	-	-	0.13 (0.01-1.32)
Adjusted HR†	-	-	-	-	-	-	0.14 (0.01-1.69)

HDL-C, high-density lipoprotein cholesterol; HR, hazard ratio; MACEs, major adverse cardiovascular events; MI, myocardial infarction.

* model 1: age, sex

† model 2: age, sex, body mass index, smoking, drinking, hypertension, history of dyslipidemia, history of stroke, history of atrial fibrillation, history of coronary heart disease, medication history of antiplatelet agents, medication history of lipid-lowering drugs, low-density lipoprotein cholesterol, triglycerides, National Institutes of Health Stroke Scale on admission, TOAST, use of antiplatelet agents at discharge, use of anticoagulant agents at discharge, and use of statins at discharge.

Supplementary Table S3 Sensitivity Analysis for the Association between HDL-C and Adverse Outcomes

Outcomes	HDL-C quintiles, mmol/L					P for trend	Each 1-mmol/L increase in HDL-C
	Q1, <0.71 mmol/L	Q2, 0.71-0.83 mmol/L	Q3, 0.83-0.95 mmol/L	Q4, 0.95-1.11 mmol/L	Q5, ≥1.11 mmol/L		
**6 months**							
**Recurrent stroke**							
Events, n (%)	75 (10.33)	64 (9.41)	62 (8.39)	59 (8.17)	56 (7.87)		
Incidence, per 1000 person-years	230.90	208.84	185.23	178.28	171.77		
Adjusted HR*	1.44 (1.01-2.05)	1.32 (0.92-1.90)	1.14 (0.79-1.64)	1.10 (0.76-1.58)	1.00	0.025	0.72 (0.47-1.08)
Adjusted HR†	1.50 (1.02-2.21)	1.33 (0.89-1.98)	1.16 (0.78-1.71)	1.03 (0.69-1.53)	1.00	0.022	0.66 (0.42-1.05)
**MACEs**							
Events, n (%)	78 (10.74)	67 (9.85)	68 (9.20)	62 (8.59)	58 (8.15)		
Incidence, per 1000 person-years	240.19	218.63	203.46	187.44	177.91		
Adjusted HR*	1.45 (1.03-2.05)	1.34 (0.94-1.91)	1.21 (0.85-1.72)	1.11 (0.78-1.60)	1.00	0.020	0.69 (0.46-1.04)
Adjusted HR†	1.52 (1.04-2.22)	1.36 (0.92-2.00)	1.24 (0.85-1.81)	1.03 (0.70-1.51)	1.00	0.014	0.63 (0.40-0.99)
**12 months**							
**Recurrent stroke**							
Events, n (%)	89 (12.26)	80 (11.76)	81 (10.96)	69 (9.56)	68 (9.55)		
Incidence, per 1000 person-years	140.29	133.89	123.47	105.93	106.31		
Adjusted HR*	1.42 (1.03-1.95)	1.37 (0.98-1.90)	1.23 (0.89-1.70)	1.05 (0.75-1.48)	1.00	0.012	0.73 (0.50-1.07)
Adjusted HR†	1.53 (1.07-2.17)	1.40 (0.98-2.00)	1.29 (0.92-1.83)	1.02 (0.71-1.47)	1.00	0.006	0.65 (0.43-0.98)
**MACEs**							

Outcomes	HDL-C quintiles, mmol/L<br>Q1, <0.71 mmol/L	Q2, 0.71-0.83 mmol/L	Q3, 0.83-0.95 mmol/L	Q4, 0.95-1.11 mmol/L	Q5, ≥1.11 mmol/L	P for trend	Each 1-mmol/L increase in HDL-C
Events, n (%)	92 (12.67)	84 (12.35)	88 (11.91)	73 (10.11)	72 (10.11)		
Incidence, per 1000 person-years	145.27	140.63	134.75	112.25	112.57		
Adjusted HR*	1.37 (1.00-1.88)	1.34 (0.98-1.85)	1.26 (0.92-1.72)	1.05 (0.76-1.46)	1.00	0.017	0.73 (0.51-1.06)
Adjusted HR†	1.46 (1.03-2.06)	1.36 (0.96-1.93)	1.32 (0.94-1.84)	1.00 (0.70-1.42)	1.00	0.009	0.65 (0.44-0.98)

HDL-C, high-density lipoprotein cholesterol; MACEs, major adverse cardiovascular events.

* model 1: age, sex

† model 2: age, sex, body mass index, smoking, drinking, hypertension, history of dyslipidemia, history of stroke, history of atrial fibrillation, history of coronary heart disease, medication history of antiplatelet agents, medication history of lipid-lowering drugs, low-density lipoprotein cholesterol, triglycerides, National Institutes of Health Stroke Scale on admission, TOAST, use of antiplatelet agents at discharge, use of anticoagulant agents at discharge, and use of statins at discharge.

Supplementary Table S4 Multivariable Analysis of Association between Low Levels of HDL-C and Adverse Outcomes within 1 year Compared with Normal Levels of HDL-C

	Events, n (%)	Model 1*: HR (95% CI)	P value	Model 2†: HR (95% CI)	P value
6 months					
Recurrent stroke	243 (9.52)	1.29 (1.02-1.63)	0.038	1.40 (1.07-1.82)	0.014
MACEs	259 (10.15)	1.33 (1.06-1.68)	0.016	1.45 (1.12-1.88)	0.005
12 months					
Recurrent stroke	299 (11.72)	1.34 (1.08-1.67)	0.008	1.47 (1.16-1.87)	0.002
MACEs	318 (12.46)	1.35 (1.09-1.67)	0.005	1.47 (1.17-1.86)	0.001

HDL-C, high-density lipoprotein cholesterol; MACEs, major adverse cardiovascular events.
* model 1: age, sex
† model 2: age, sex, body mass index, smoking, drinking, hypertension, history of dyslipidemia, history of stroke, history of atrial fibrillation, history of coronary heart disease, medication history of antiplatelet agents, medication history of lipid-lowering drugs, low-density lipoprotein cholesterol, triglycerides, National Institutes of Health Stroke Scale on admission, TOAST, use of antiplatelet agents at discharge, use of anticoagulant agents at discharge, and use of statins at discharge.

Supplementary Table S5.1 Subgroup Analysis for the Association between HDL-C and Recurrent Stroke at 6 Months

Subgroup	Events (%)	HDL-C quintiles, mmol/L					P for trend	P for interaction
		Q1, <0.71 mmol/L	Q2, 0.71-0.83 mmol/L	Q3, 0.83-0.95 mmol/L	Q4, 0.95-1.11 mmol/L	Q5, ≥1.11 mmol/L		
**Age**								
<65 years	171 (7.91)	1.43 (0.81-2.53)	1.40 (0.81-2.43)	1.20 (0.69-2.08)	1.06 (0.60-1.86)	1.00	0.131	0.960
≥65 years	173 (10.41)	1.80 (1.08-3.01)	1.63 (0.95-2.78)	1.18 (0.70-2.01)	1.12 (0.66-1.89)	1.00	0.015	
**Sex**								
Men	217 (8.62)	1.56 (0.94-2.59)	1.54 (0.93-2.55)	1.09 (0.64-1.85)	1.03 (0.60-1.77)	1.00	0.027	0.921
Women	127 (9.72)	1.52 (0.83-2.78)	1.33 (0.71-2.50)	1.43 (0.82-2.48)	1.15 (0.66-1.99)	1.00	0.133	
**Smoking**								
No	250 (9.32)	1.62 (1.05-2.50)	1.61 (1.04-2.47)	1.15 (0.74-1.77)	1.07 (0.69-1.65)	1.00	0.011	0.717
Yes	94 (8.22)	1.59 (0.71-3.53)	1.29 (0.57-2.90)	1.42 (0.65-3.10)	1.21 (0.54-2.72)	1.00	0.265	
**Drinking**								
No	287 (8.68)	1.44 (0.95-2.18)	1.43 (0.95-2.15)	1.10 (0.73-1.67)	1.08 (0.72-1.62)	1.00	0.044	0.923
Yes	57 (11.03)	2.68 (0.93-7.72)	2.27 (0.76-6.77)	2.15 (0.75-6.20)	1.24 (0.38-4.02)	1.00	0.036	
**Hypertension**								
No	21 (6.52)	0.22 (0.02-2.28)	1.28 (0.31-5.34)	0.69 (0.13-3.53)	0.99 (0.22-4.50)	1.00	0.420	0.517
Yes	323 (9.22)	1.71 (1.16-2.53)	1.53 (1.03-2.27)	1.25 (0.85-1.85)	1.13 (0.76-1.68)	1.00	0.003	
**NIHSS score on admission**								
0-4	147 (8.03)	2.19 (1.18-4.05)	1.85 (0.99-3.46)	1.64 (0.90-3.01)	1.74 (0.96-3.15)	1.00	0.017	0.325
≥4	197 (9.88)	1.31 (0.80-2.13)	1.35 (0.83-2.19)	1.01 (0.62-1.66)	0.83 (0.50-1.39)	1.00	0.118	
**Ischemic stroke subtype**								
Large-artery atherosclerosis	117 (11.05)	1.27 (0.63-2.55)	1.58 (0.81-3.07)	1.35 (0.70-2.63)	1.19 (0.60-2.37)	1.00	0.346	0.682
Cardiac	23 (10.85)	0.54 (0.12-2.53)	0.61 (0.10-3.60)	0.37 (0.07-1.82)	0.33 (0.07-1.50)	1.00	0.368	

**embolism**							
**Small-vessel occlusion**	51 (5.91)	2.01 (0.69-5.84)	3.27 (1.20-8.88)	1.51 (0.52-4.35)	1.21 (0.40-3.68)	1.00	0.052
**Other**	153 (9.05)	2.03 (1.17-3.54)	1.34 (0.73-2.45)	1.27 (0.71-2.26)	1.25 (0.71-2.19)	1.00	0.018

HDL-C, high-density lipoprotein cholesterol; NIHSS, National Institutes of Health Stroke Scale.

Supplementary Table S5.2 Subgroup Analysis for the Association between HDL-C and MACEs at 6 Months

Subgroup	Events (%)	HDL-C quintiles, mmol/L					P for trend	P for interaction
		Q1, <0.71 mmol/L	Q2, 0.71-0.83 mmol/L	Q3, 0.83-0.95 mmol/L	Q4, 0.95-1.11 mmol/L	Q5, ≥1.11 mmol/L		
**Age**								
**<65 years**	177 (8.19)	1.37 (0.78-2.38)	1.38 (0.81-2.35)	1.11 (0.65-1.89)	0.97 (0.56-1.69)	1.00	0.138	0.910
**≥65 years**	186 (11.19)	1.94 (1.17-3.21)	1.74 (1.03-2.95)	1.45 (0.88-2.40)	1.19 (0.71-1.98)	1.00	0.005	
**Sex**								
**Men**	231 (9.18)	1.57 (0.96-2.57)	1.53 (0.94-2.49)	1.14 (0.69-1.88)	0.95 (0.56-1.61)	1.00	0.014	0.851
**Women**	132 (10.10)	1.55 (0.85-2.83)	1.46 (0.79-2.69)	1.56 (0.91-2.67)	1.25 (0.73-2.13)	1.00	0.094	
**Smoking**								
**No**	263 (9.81)	1.66 (1.08-2.54)	1.72 (1.13-2.62)	1.20 (0.79-1.84)	1.09 (0.71-1.67)	1.00	0.005	0.505
**Yes**	100 (8.75)	1.55 (0.72-3.31)	1.15 (0.53-2.54)	1.46 (0.70-3.06)	1.08 (0.49-2.37)	1.00	0.253	
**Drinking**								
**No**	306 (9.25)	1.49 (0.99-2.22)	1.48 (0.99-2.21)	1.19 (0.80-1.77)	1.07 (0.72-1.59)	1.00	0.021	0.952
**Yes**	57 (11.03)	2.68 (0.93-7.72)	2.27 (0.76-6.77)	2.15 (0.75-6.20)	1.24 (0.38-4.02)	1.00	0.036	
**Hypertension**								
**No**	22 (6.83)	0.44 (0.07-2.88)	1.27 (0.31-5.18)	0.63 (0.13-3.19)	0.93 (0.20-4.23)	1.00	0.611	0.686
**Yes**	341 (9.74)	1.72 (1.17-2.51)	1.57 (1.07-2.31)	1.33 (0.91-1.94)	1.12 (0.76-1.65)	1.00	0.002	
**NIHSS score on admission**								
**0-4**	152 (8.30)	1.97 (1.09-3.56)	1.67 (0.91-3.05)	1.44 (0.80-2.60)	1.54 (0.87-2.74)	1.00	0.032	0.491
**≥4**	211 (10.59)	1.45 (0.90-2.34)	1.48 (0.92-2.38)	1.22 (0.76-1.95)	0.86 (0.52-1.43)	1.00	0.035	
**Ischemic stroke subtype**								
**Large-artery atherosclerosis**	125 (11.80)	1.23 (0.62-2.44)	1.62 (0.85-3.09)	1.49 (0.79-2.82)	1.26 (0.65-2.43)	1.00	0.376	0.460
**Cardiac**	23 (10.85)	0.54 (0.12-2.53)	0.61 (0.10-3.60)	0.37 (0.07-1.82)	0.33 (0.07-1.50)	1.00	0.368	

**embolism**							
**Small-vessel occlusion**	54 (6.26)	1.97 (0.68-5.73)	3.66 (1.37-9.76)	1.66 (0.59-4.68)	1.21 (0.40-3.67)	1.00	0.040
**Other**	161 (9.53)	2.17 (1.27-3.70)	1.33 (0.74-2.42)	1.31 (0.75-2.29)	1.18 (0.67-2.05)	1.00	0.006

HDL-C, high-density lipoprotein cholesterol; MACEs, major adverse cardiovascular events; NIHSS, National Institutes of Health Stroke Scale.

Supplementary Table S5.3 Subgroup Analysis for the Association between HDL-C and Recurrent Stroke at 12 Months

Subgroup	Events (%)	HDL-C quintiles, mmol/L					P for trend	P for interaction
		Q1, <0.71 mmol/L	Q2, 0.71-0.83 mmol/L	Q3, 0.83-0.95 mmol/L	Q4, 0.95-1.11 mmol/L	Q5, ≥1.11 mmol/L		
**Age**								
**<65 years**	206 (9.53)	1.50 (0.89-2.53)	1.50 (0.91-2.48)	1.25 (0.76-2.07)	1.18 (0.71-1.95)	1.00	0.077	0.948
**≥65 years**	212 (12.76)	1.72 (1.08-2.75)	1.64 (1.01-2.65)	1.37 (0.87-2.18)	1.05 (0.65-1.69)	1.00	0.008	
**Sex**								
**Men**	267 (10.61)	1.58 (1.01-2.49)	1.56 (1.00-2.45)	1.13 (0.71-1.81)	1.10 (0.68-1.76)	1.00	0.014	0.618
**Women**	151 (11.55)	1.56 (0.89-2.74)	1.44 (0.81-2.57)	1.69 (1.03-2.77)	1.08 (0.64-1.82)	1.00	0.052	
**Smoking**								
**No**	307 (11.45)	1.55 (1.04-2.30)	1.55 (1.05-2.29)	1.30 (0.89-1.89)	1.06 (0.71-1.56)	1.00	0.008	0.970
**Yes**	111 (9.71)	1.99 (0.93-4.25)	1.77 (0.83-3.78)	1.59 (0.74-3.42)	1.47 (0.68-3.20)	1.00	0.069	
**Drinking**								
**No**	354 (10.70)	1.52 (1.05-2.22)	1.48 (1.02-2.15)	1.26 (0.87-1.82)	1.13 (0.79-1.64)	1.00	0.012	0.899
**Yes**	64 (12.38)	2.30 (0.86-6.12)	2.39 (0.91-6.26)	2.04 (0.79-5.24)	0.99 (0.33-3.01)	1.00	0.032	
**Hypertension**								
**No**	25 (7.76)	0.18 (0.02-1.91)	1.75 (0.47-6.57)	0.56 (0.11-2.85)	1.18 (0.29-4.90)	1.00	0.464	0.232
**Yes**	393 (11.22)	1.73 (1.21-2.46)	1.54 (1.08-2.20)	1.38 (0.98-1.96)	1.13 (0.79-1.62)	1.00	<0.001	
**NIHSS score on admission**								
**0-4**	181 (9.89)	1.98 (1.14-3.41)	1.88 (1.09-3.24)	1.71 (1.01-2.89)	1.43 (0.83-2.44)	1.00	0.008	0.561
**≥4**	237 (11.89)	1.39 (0.89-2.19)	1.39 (0.89-2.18)	1.13 (0.72-1.77)	0.98 (0.62-1.56)	1.00	0.070	
**Ischemic stroke subtype**								
**Large-artery atherosclerosis**	140 (13.22)	1.37 (0.73-2.59)	1.78 (0.97-3.24)	1.51 (0.83-2.76)	1.25 (0.67-2.33)	1.00	0.175	0.640
**Cardiac**	29 (13.68)	0.87 (0.23-3.30)	0.53 (0.09-3.06)	0.79 (0.21-2.89)	0.29 (0.07-1.30)	1.00	0.866	

**embolism**							
**Small-vessel occlusion**	69 (8.00)	1.08 (0.45-2.63)	1.95 (0.88-4.30)	1.07 (0.47-2.45)	1.06 (0.46-2.44)	1.00	0.433
**Other**	180 (10.65)	2.23 (1.33-3.74)	1.55 (0.89-2.70)	1.44 (0.85-2.45)	1.25 (0.74-2.13)	1.00	0.002

HDL-C, high-density lipoprotein cholesterol; NIHSS, National Institutes of Health Stroke Scale.

Supplementary Table S5.4 Subgroup Analysis for the Association between HDL-C and MACEs at 12 Months

Subgroup	Events (%)	HDL-C quintiles, mmol/L					P for trend	P for interaction
		Q1, <0.71 mmol/L	Q2, 0.71-0.83 mmol/L	Q3, 0.83-0.95 mmol/L	Q4, 0.95-1.11 mmol/L	Q5, ≥1.11 mmol/L		
**Age**								
**<65 years**	217 (10.04)	1.32 (0.80-2.19)	1.40 (0.87-2.25)	1.14 (0.71-1.84)	1.01 (0.62-1.66)	1.00	0.132	0.830
**≥65 years**	226 (13.60)	1.79 (1.13-2.83)	1.69 (1.05-2.71)	1.57 (1.01-2.45)	1.12 (0.70-1.79)	1.00	0.004	
**Sex**								
**Men**	288 (11.44)	1.48 (0.96-2.28)	1.48 (0.97-2.26)	1.16 (0.75-1.79)	0.98 (0.62-1.54)	1.00	0.017	0.687
**Women**	155 (11.86)	1.58 (0.90-2.77)	1.54 (0.87-2.72)	1.74 (1.06-2.84)	1.16 (0.69-1.93)	1.00	0.041	
**Smoking**								
**No**	324 (12.09)	1.52 (1.03-2.24)	1.61 (1.10-2.35)	1.31 (0.91-1.90)	1.06 (0.72-1.55)	1.00	0.007	0.915
**Yes**	119 (10.41)	1.71 (0.84-3.47)	1.42 (0.69-2.91)	1.55 (0.77-3.12)	1.19 (0.57-2.49)	1.00	0.113	
**Drinking**								
**No**	378 (11.43)	1.47 (1.02-2.11)	1.47 (1.02-2.10)	1.29 (0.91-1.83)	1.09 (0.76-1.56)	1.00	0.013	0.888
**Yes**	65 (12.57)	2.22 (0.83-5.90)	2.29 (0.88-5.99)	2.15 (0.85-5.46)	0.98 (0.33-2.96)	1.00	0.038	
**Hypertension**								
**No**	26 (8.07)	0.37 (0.06-2.37)	1.73 (0.47-6.39)	0.54 (0.11-2.66)	1.13 (0.27-4.66)	1.00	0.627	0.326
**Yes**	417 (11.91)	1.63 (1.16-2.30)	1.52 (1.07-2.15)	1.41 (1.01-1.97)	1.09 (0.77-1.54)	1.00	0.001	
**NIHSS score on admission**								
**0-4**	188 (10.27)	1.74 (1.03-2.95)	1.67 (0.99-2.82)	1.49 (0.90-2.47)	1.30 (0.78-2.17)	1.00	0.024	0.809
**≥4**	255 (12.79)	1.44 (0.92-2.23)	1.46 (0.94-2.26)	1.31 (0.86-2.01)	0.97 (0.62-1.52)	1.00	0.034	
**Ischemic stroke subtype**								
**Large-artery**	150 (14.16)	1.33 (0.71-2.48)	1.84 (1.02-3.30)	1.68 (0.94-2.99)	1.29 (0.71-2.37)	1.00	0.183	0.366

**atherosclerosis**							
**Cardiac embolism**	31 (14.62)	0.76 (0.20-2.80)	0.37 (0.06-2.16)	0.77 (0.22-2.67)	0.31 (0.08-1.24)	1.00	0.687
**Small-vessel occlusion**	72 (8.34)	1.07 (0.44-2.59)	2.15 (0.99-4.68)	1.15 (0.51-2.59)	1.05 (0.46-2.43)	1.00	0.365
**Other**	190 (11.24)	2.10 (1.29-3.44)	1.39 (0.81-2.37)	1.34 (0.80-2.23)	1.09 (0.65-1.82)	1.00	0.002

HDL-C, high-density lipoprotein cholesterol; MACEs, major adverse cardiovascular events; NIHSS, National Institutes of Health Stroke Scale.

Supplementary Figure

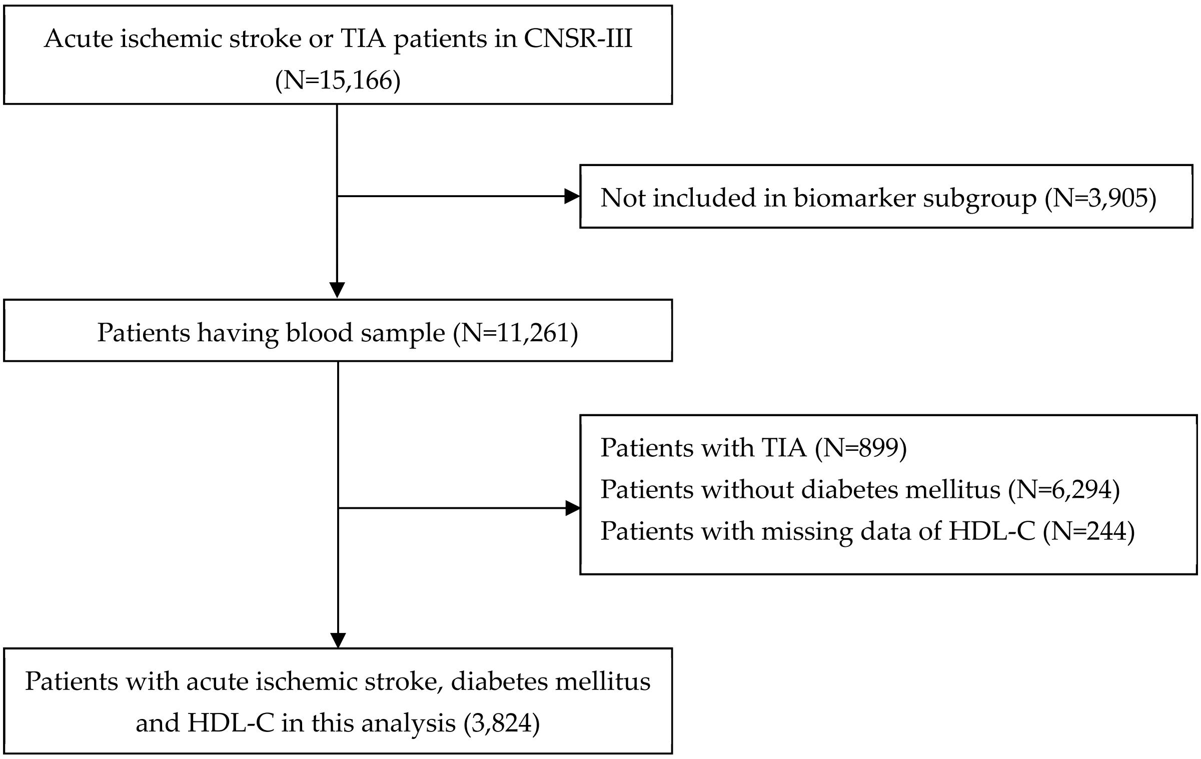

Figure S1 Flow Chart of the Study Population

TIA, transient ischemic attack; CNSR-III, Third China National Stroke Registry; HDL-C, high-density lipoprotein cholesterol
